# Influence of Magnetron Sputtering-Deposited Niobium Nitride Coating and Its Thermal Oxidation on the Properties of AISI 316L Steel in Terms of Its Medical Applications

**DOI:** 10.3390/ma16216890

**Published:** 2023-10-27

**Authors:** Tomasz Borowski, Justyna Rospondek, Marek Betiuk, Bogusława Adamczyk-Cieślak, Maciej Spychalski

**Affiliations:** 1 Faculty of Materials Science and Engineering, Warsaw University of Technology, 02-507 Warsaw, Poland; 2 Łukasiewicz Research Network—Warsaw Institute of Technologies, 01-796 Warsaw, Poland

**Keywords:** NbN, Nb_2_O_5_, magnetron sputtering, oxidising, SBF, microstructure, corrosion, surface engineering

## Abstract

An NbN coating was produced on AISI 316L steel using reactive DC magnetron sputtering. The effects of oxidation of the NbN coating in air on the microstructure, mechanical properties, corrosion resistance, contact angle and bioactivity were investigated. Phase composition was determined using X-ray diffraction (XRD), the coatings’ cross-sectional microstructure and thickness including surface morphology using a scanning electron microscope (SEM), microhardness via the Vickers method, corrosion by means of a potentiodynamic polarisation test in Ringer’s solution and bioactivity by observation in an SBF solution, while the contact angle was studied using a goniometer. The NbN coating and the oxidised coating were shown to demonstrate a Ca/P ratio close to that of hydroxyapatite, as well as increased microhardness and corrosion resistance. The best combination of mechanical, corrosion, bioactivity and hydrophilic properties was demonstrated by the air oxidised NbN coating, which featured an orthorhombic Nb_2_O_5_ structure in the top, surface layer.

## 1. Introduction

Magnetron sputtering is commonly used to produce metallic [1] and intermetallic [2] as well as ceramic [3,4] coatings. Transition metal nitrides are the most commonly used materials due to their high wear resistance and corrosion resistance in aggressive environments [5]. One such material is niobium nitride, NbN, which was widely studied in the early years more for its high temperature superconductivity properties [6,7] than for its high mechanical and chemical properties (hardness, toughness, wear resistance, good thermal stability, excellent corrosion resistance and high chemical inertness) [8,9,10]. NbN coatings are usually produced by means of unbalanced magnetron sputtering [11,12,13], high power impulse magnetron sputtering (HPIMS) [14,15], double-cathode glow discharge [12], multi-arc ion plating [16] and reactive pulsed laser deposition [8]. Ceramic coatings characterised by very good anti-corrosion and anti-wear properties are successfully used on austenitic steels [17,18], which, in turn, show relatively low hardness and friction wear resistance, and limited corrosion resistance in aggressive environments, especially those containing chlorides [19]. The application of NbN coatings on AISI 316L steel, which is still commonly used in medicine, can provide it with improved wear and corrosion resistance, as well as improved biological properties. Up to now, there have been only a limited number of reports concerning the corrosion and wear properties of NbN coatings [20], and there is a particular lack of data on their biological properties. Niobium oxides constitute another interesting group of materials in light of their anti-corrosion, anti-wear and biomedical applications [21,22,23]. The formation of a bioceramic oxide coating on the surface of an implant is usually a more effective method for increasing corrosion resistance and biocompatibility. Of the many oxide materials currently under investigation (TiO_2_, SiO_2_ and ZrO_2_), niobium oxide Nb_2_O_5_ is the preferred coating material due to its extremely high corrosion resistance, thermodynamic stability, and high biocompatibility [24]. Moreover, some studies have shown that osteoblast adhesion and proliferation on Nb_2_O_5_ are comparable to TiO_2_ [25]. On the other hand, study [26] showed that cp-Ti titanium coated with niobium oxide showed increased migration and adhesion of MC3T3-E1 cells compared to uncoated titanium. Another study [27] saw better bone tissue adhesion to niobium than to titanium. Furthermore, it has been reported that materials based on Nb are hypoallergenic, as the metal is safe and is tolerated by the human body [28]. Nevertheless, there are relatively few studies on the bioactivity and biocompatibility of Nb_2_O_5_ coatings. Combining the process of magnetron production of NbN coatings and thermal oxidation to produce Nb_2_O_5_ niobium oxide on the surface of an NbN coating can result in a material with high hardness, corrosion resistance and better bioactivity compared to uncoated AISI 316L steel.

The purpose of this study was to analyse the microstructure, corrosion resistance in Ringer’s solution, contact angle and bioactivity in SBF solution of NbN coatings and oxidised NbN coatings with an Nb_2_O_5_ top layer produced on AISI 316L steel in terms of their medical applications, and to compare the properties of the produced coatings and layers to steel in its initial state.

## 2. Materials and Methods

### 2.1. Specimen Preparation

**Figure 1:**
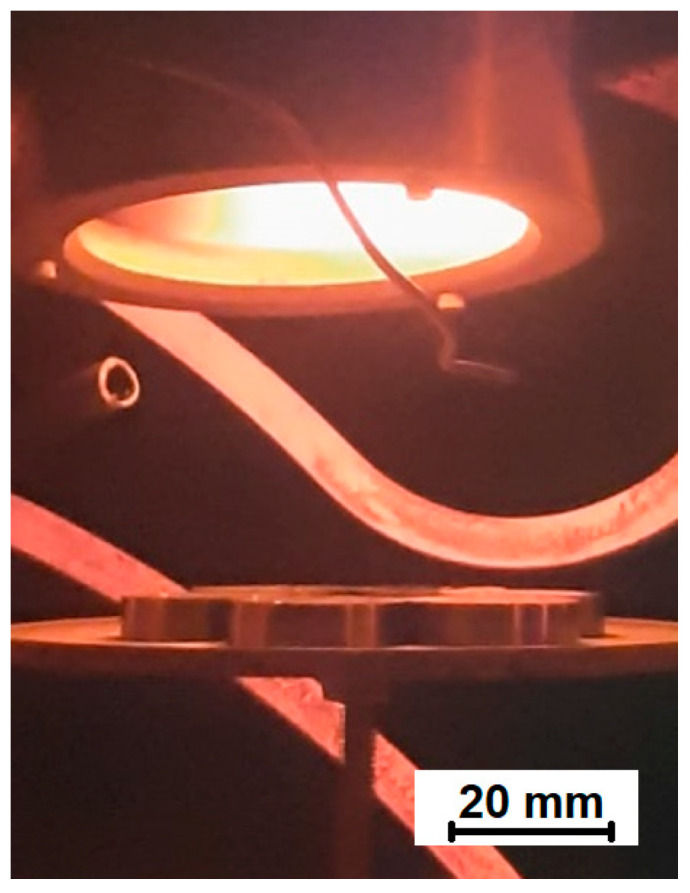
Magnetron and sputtered AISI 316L steel samples.

The AISI 316L steel specimens used for the study took the form of ϕ25 × 6 mm discs. They were first ground using 240- to 1200-grit sandpaper, then degreased in acetone using an ultrasonic cleaner. The samples prepared in this way were coated with NbN by magnetron sputtering, after which some were oxidised in a furnace (Nabertherm, Lilienthal, Germany, model RS 80/300/13) under an atmosphere of air. The coating sputtering process was carried out by magnetron sputtering using a WU-1B vacuum station (National Academy of Sciences of Belarus, Minsk) with a 0.4 m^3^ chamber and an 8000 L/sec diffusion pump. The vacuum chamber was equipped with a magnetron plasma source, a circular flat magnetron (Kurt J. Lesker, Jefferson Hills, PA, USA, type-TORUS 3HVm) with a 3” diameter Nb (99.95% pure) cathode (Kurt J. Lesker), a Pfeiffer Vacuum PTR 91 vacuum meter (Asslar, Germany) and resistive heat emitters. The magnetron plasma was excited by means of a Dora’s MSS-10 power supply (Wilczyce, Poland) with a DC pulsed current of 100 kHz. The initial ion etching of the niobium target lasted 3 min and was carried out in an Ar atmosphere at 1000 V, 3.22 A, 1.2 kW, 10 Pa and 360 °C substrate temperature. The target was placed over the samples and the distance between them was 70 mm (Figure 1). The next step was the generation of NbN coatings, which was carried out in an atmosphere of Ar and N_2_ at a ratio of 5:1 (20% N_2_) for 30 min, at 0.8 Pa, 4.9 A, 2 kW, −80 V substrate polarisation and 438 °C deposition temperature. The parameters were selected to produce cubic δ-NbN, which exhibits higher oxidation kinetics than hexagonal β-Nb_2_N [29]. The process had to be performed below 450 °C in an N_2_ atmosphere to avoid the precipitation of chromium nitrides in the steel’s top surface layer, which could reduce resistance to intergranular corrosion [19]. A Nabertherm tube furnace was used to oxidise selected NbN-coated samples in air at atmospheric pressure for 4 h, at a heat rate of 10 °C/min and at 480 °C. According to Qi et al. [29], the best oxidation kinetics of the δ-NbN coating were obtained at a temperature close to 500 °C. To avoid the formation of chromium carbides in austenitic steel, which are also detrimental to intergranular corrosion resistance [19], the oxidation process had to be performed at a temperature lower than 500 °C.

### 2.2. Microstructural and Hardness Analysis

The samples used for the microstructure study were cut along their diameter, then encapsulated in epoxy resin, ground with 80 to 1200 sandpaper and polished using a diamond slurry with a grain size of 1 μm. Etching of the samples was carried out using a reagent consisting of 50% HCl + 25% HNO_3_ + 25% H_2_O. The cross-sectional microstructure was studied using a Hitachi’s S-3500N scanning electron microscope (SEM) (Tokyo, Japan) with a secondary electron (SE) detector at an accelerating voltage of 15 kV. Microhardness tests were carried out via Shimadzu’s Vickers HMV-G hardness tester (Kyoto, Japan) under 50 g and 100 g loads. Seven indentations were made on each sample under each load.

### 2.3. Phase and Chemical Composition Analysis

The phase composition of the samples was studied using the X-ray diffraction (XRD) method. A D8 Advance X-ray diffractometer (Bruker AXS, Karlsruhe, Germany) was used for the study. The lamp included in the testing apparatus was made of copper, while the radiation length of CuKα was λ = 0.154056 nm. The analyses were carried out at 40 kV, 40 mA, a step of Δ2θ = 0.05° and a count time of 3 sec. The angular range of 2θ stretched from 15° to 100° for the NbN coating and it was established in Bragg–Brentano geometry. The study of the thin oxide film produced on the NbN coating was carried out by means of grazing incidence angle diffraction at a constant X-ray beam angle of 2° over an angular range of 2θ from 10° to 100°. The recorded diffraction patterns were analysed using Brucker’s EVA software, version V3.0. An analysis of the chemical composition in two different areas (3 measurements each) on the surface of the oxidised NbN coating was also carried out, together with imaging of the distribution map of oxygen, nitrogen, niobium and iron on its cross-sections, using a ThermoNoran’s energy dispersive X-ray spectroscope (EDS) (Madison, WI, USA) equipped with a Hitachi S-3500N scanning electron microscope. The measurements were carried out at a voltage of 15 kV.

### 2.4. Surface Topography Analysis

Surface roughness tests were conducted using Veeco’s Wyko NT9300 profilometer (Plainview, NY, USA). The parameters Ra (arithmetic mean deviation of the profile from the mean line) and Rt (total profile height, i.e., sum of the highest elevation point and the lowest point of the profile) were determined using Vision software, version 4.10. Five surface roughness measurements were taken for each sample, which made it possible to calculate the average roughness value parameters. The surface morphology of the prepared samples was studied using a Hitachi S-3500N scanning electron microscope (SEM) with a secondary electron (SE) detector at an accelerating voltage of 15 kV.

### 2.5. Corrosion Measurements

Corrosion resistance of AISI 316L steel at baseline and with an NbN coating and oxidised layer was measured in Ringer’s solution at a temperature of 37 °C by means of the potentiodynamic method using an Atlas-Sollich’s 0531 EU&IA device (Rębiechowo, Poland). A three-electrode setup was used in the tests comprising a test electrode, an Ag/AgCl electrode, i.e., the reference electrode and a platinum gauze counter electrode. The tests were performed in an electrochemical vessel, in which the surfaces of the samples were pressed against a hole with a gasket limiting the surface area of the samples to 0.33 cm^2^. Prior to testing, the samples were held in the measurement array for 2 h to stabilise them and determine their open circuit potential (OCP). Polarisation resistance was then tested using the Stern method by polarising the test material from a potential of 10 mV below to 10 mV above the determined OCP at a sweep rate of 0.2 mV/s. The R_pol_ polarisation resistance was determined from the E = f(i) dependence. The anodic polarisation curves of the tested materials were then recorded using the potentiodynamic polarisation method. The samples were polarised from a potential 0.25 V lower than the OCP to a potential of 1.5 V. In the potential range of ±0.25 V from the OCP a polarisation rate of 0.2 mV/s was used, whereas, in the remaining potential range, the rate was 0.7 mV/s. For each variant, at least three measurements were made. E_pit_ pitting potentials and i_pass_ passive state current density at a potential of 100 mV were read from the polarisation curves. The i_corr_ corrosion current density and E_corr_ corrosion potential were determined using the Tafel extrapolation method. After potentiodynamic tests, the samples’ surfaces were observed again using a Nikon Eclypse LV150N optical microscope (Nikon Instruments, Melville, NY, USA).

### 2.6. Bioactivity and Wettability Analysis

Bioactivity tests of coatings and layers were carried out using an SBF solution prepared according to Kokubo’s guidelines [30]. Samples of AISI 316L steel in the initial state, NbN-coated steel and oxidised NbN-coated steel were placed in separate sterile containers. The solution was then added to the containers to keep the entire surface of the samples in contact with the ions in the solution. The sealed sample containers were then placed in a heat chamber for 2 weeks at 37 °C. During the test, the solution was replenished every 2 days. After 2 weeks, the surfaces of the samples were examined using a scanning electron microscope and an energy-dispersive X-ray EDS spectrometer. A total of five measurements of the chemical composition of the surface of each sample were carried out. Contact angle examinations were carried out using a RAME-HART 90-U3-PRO goniometer (ramé-hart instrument, Succasunna, NJ, USA) equipped with a CCD camera, an adjustable table, a syringe for manual droplet dispensing and DROPImage software, version 3.22.06.0. The liquid used in the study consisted of distilled water. A total of five measurements were taken on each sample.

## 3. Results and Discussion

**Figure 2:**
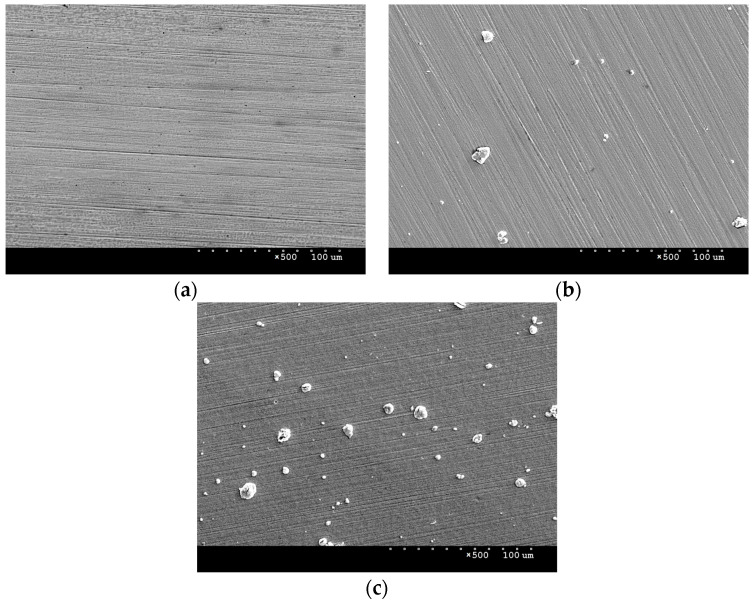
SEM images of the samples’ surfaces: AISI 316L steel at baseline (**a**), deposited NbN coating (**b**) and oxidised NbN coating (**c**); magnification ×500.

**Figure 3:**
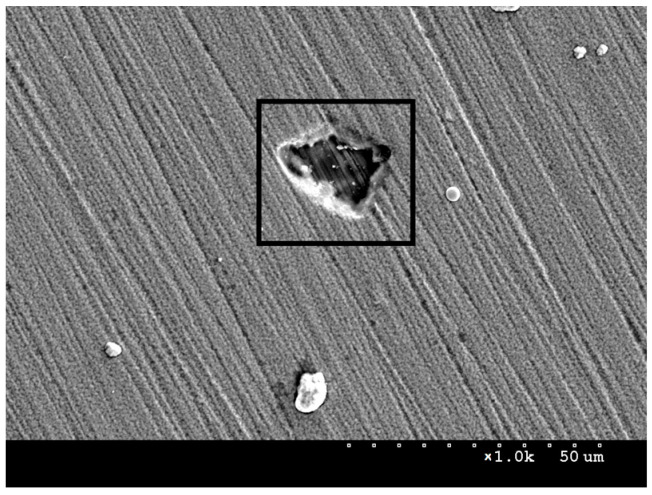
SEM image of AISI 316L steel surface with deposited NbN coating and a crater indicated by the black frame, magnification ×1000.

SEM scanning electron microscope observations were carried out to analyse the surface morphology of the samples. Figure 2a presents an image of the surface of AISI 316L steel in its initial state. The image shows scratches remaining after grinding, as well as darker areas of small size, which most likely represent residual impurities that could not be removed during degreasing. From the SEM images, it can be concluded that the coating and layer produced are continuous, with no major losses or inhomogeneities visible. Figure 2b,c shows images of the surface of the steel with the deposited NbN coating and the NbN coating after annealing in air. Bright niobium nitrides can be observed, which in places form agglomerates of about 10–20 μm in size. They are often observed on the surface of materials after magnetron sputtering processes. During microscopic observations, craters similar in size to niobium nitride agglomerates were observed, which may indicate that they are weakly bound to the surface and can be removed under stress (Figure 3) in a hard layer.

**Figure 4:**
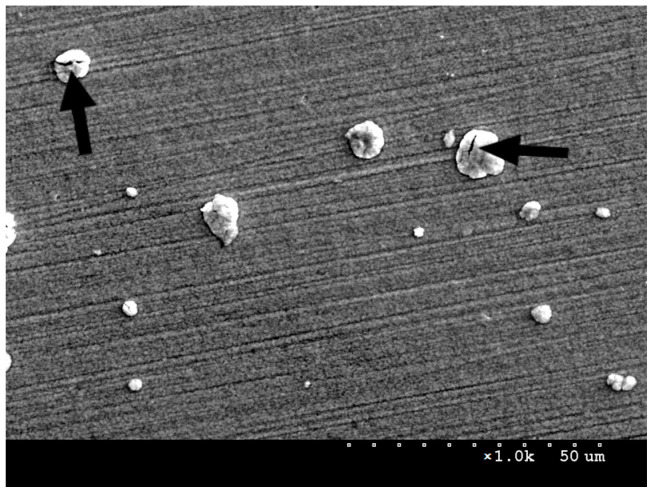
SEM image of AISI 316L steel surface with oxidised NbN coating showing nitride and niobium oxide agglomerates on the surface, magnification ×1000. The black arrows indicate cracks in the agglomerates.

In addition to niobium nitrides, smaller, bright oxides can be observed on the surface of the oxidised NbN coating (Figure 2c and Figure 4). In addition, it was noted that, following oxidation, new agglomerates are formed where the nitrides are detached from the surface. This indicates a possibility that the oxides partly filled the discontinuities in the NbN coating during the oxidation process. Compared to the NbN coating, a larger size distribution and higher agglomerate density are visible, which results from the surface accommodating both nitrides and oxides. Fractures in larger sized agglomerates were also observed in the images (Figure 4), indicating that the resulting phases may exhibit high stress and brittleness.

**Figure 5:**
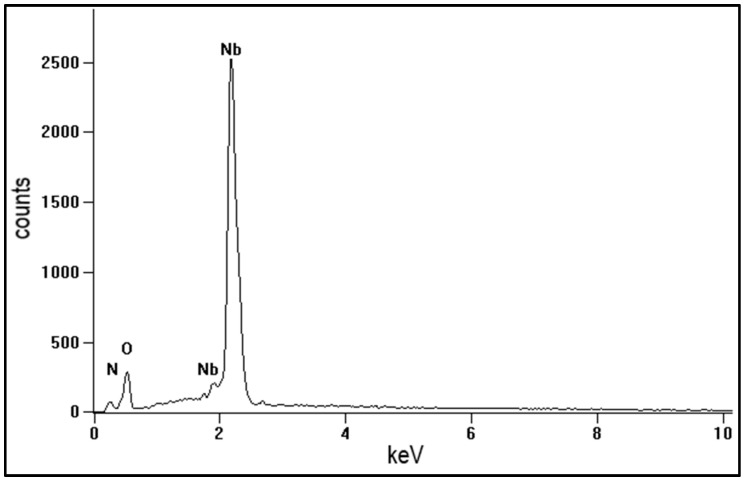
EDS spectrum obtained in the agglomerate on oxidised NbN surface.

The chemical composition (Table 1, Figure 5) was examined on the surface of the oxidised NbN coating in agglomerates and on flat surfaces of the layer outside agglomerates (Figure 4). The agglomerates were characterised by a higher oxygen content compared to the flat surface of the oxidised layer, which may explain the presence of cracks in their structure. An increase in the oxygen content may lead to the greater stress in the oxides and to their embrittlement. The presence of nitrogen may result from the depth of penetration of the electron beam, which may reach up to the area of non-oxidised NbN coating.

**Figure 6:**
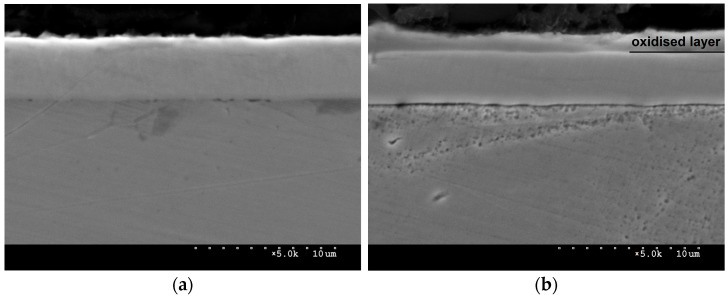
Cross-sections of NbN (**a**) and oxidised NbN (**b**) coatings on AISI 316L steel, magnification ×5000.

Layer thickness was determined by SEM observations of the metallographic specimens’ cross-sections. The niobium nitride coating shown in Figure 6a is characterised by homogeneity and continuity. Very small discontinuities in the form of pores are visible between the coating and the substrate, which may directly affect coating to substrate adhesion. The total thickness of the niobium nitride coating was about 4.5 μm. Figure 6b shows the oxidised niobium nitride coating. It is noted that an oxide layer with a smaller, varied thickness formed on the NbN coating. The total thickness of the coating was about 5.5 μm, of which the niobium nitride coating accounted for 3.8 μm, while the oxide layer accounted for 1.7 μm.

**Figure 7:**
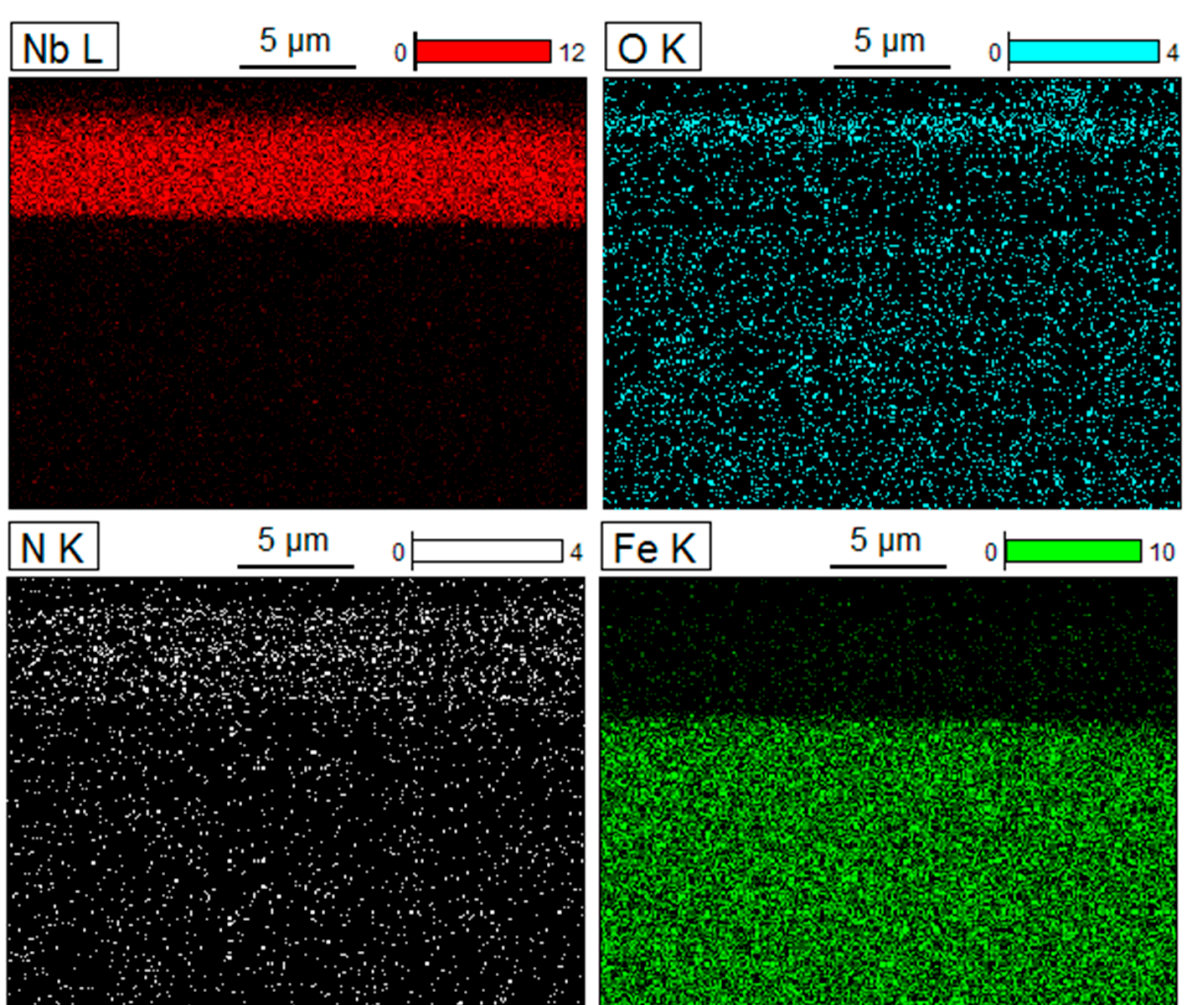
Elemental distribution on the cross-section of oxidised NbN coating.

Figure 7 shows an example of the distribution of niobium, oxygen, nitrogen and iron in the oxidised NbN layer. A uniformly oxidised layer is visible, corresponding to the surface oxide layer marked in Figure 6b.

Table 2 summarises the surface roughness of the test specimens determined using an optical profilometer. Comparison of the obtained values shows that deposition of the NbN coating caused a significant increase in roughness—a twofold increase in the Ra parameter and an almost ninefold increase in Rt vis-a-vis steel in its initial state (Table 2). This is largely due to the presence of agglomerates and discontinuities on the surface of the coating (Figure 2b and Figure 3). Oxidation of the NbN coating, on the other hand, resulted in an almost 5-fold increase in the Ra parameter and a 1.5-fold increase in Rt compared to the non-oxidised NbN coating. A clear effect of oxidation on increasing the roughness of the NbN coating can be observed. The increased roughness is mainly due to the higher number of agglomerates compared to the NbN coating (Figure 2c and Figure 4).

**Figure 8:**
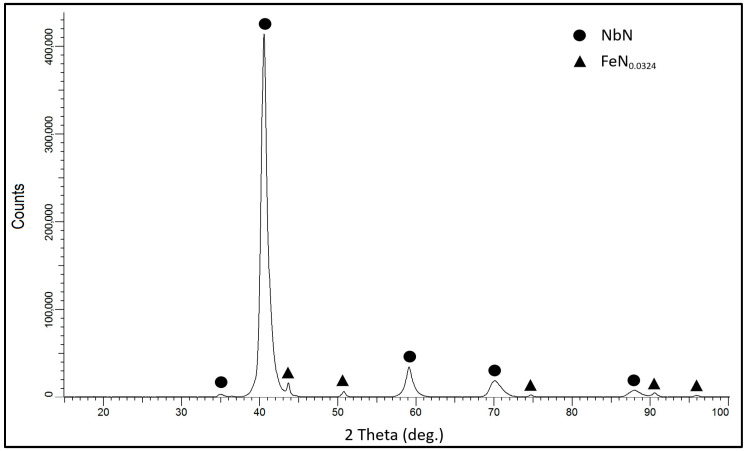
XRD pattern of NbN coating produced on AISI 316L steel.

**Figure 9:**
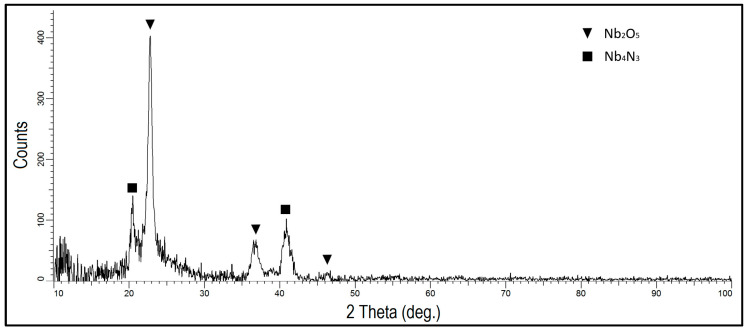
XRD pattern of oxidised NbN coating produced on AISI 316L steel.

Analysis of the NbN coating’s phase composition (Figure 8) showed the presence of cubic niobium nitride, δ-NbN, with a clearly maturing peak from the (200) plane. Low intensity peaks originating from iron nitride, FeN_0.0324_, which was formed in the surface layer of AISI 316L steel under the NbN coating, were also identified. The iron nitride most likely formed at the initial stage of coating deposition due to the presence of nitrogen in the process atmosphere. Qi et al. [29], in their study, also produced cubic niobium nitride using magnetron sputtering in a range of nitrogen contents from 10 to 40% and observed changes in the intensity of δ-NbN peaks originating from the (111) and (200) planes. As the nitrogen content in the working chamber increased, the intensities of the peaks from the planes changed from (111) in favour of (200). Niobium (V) oxide Nb_2_O_5_ with an orthorhombic structure was identified in the sample with an oxidised NbN coating (Figure 9). The Nb_4_N_3_ phase, a niobium nitride crystallising in a tetragonal lattice, was also observed in the coating structure. This shows that the niobium nitride coating under the oxide layer exhibits a different structure, meaning that the sputtered coating has a gradient phase structure after oxidising, consisting of tetragonal Nb_4_N_3_ in the outer layer and cubic δ-NbN deeper down.

It is worth noting that the Nb_4_N_3_ phase was not present in the coating before the oxidation process (Figure 8). During oxidation at 480 °C, processes may have taken place that caused nitrogen to diffuse deep into the NbN coating and form a nitrogen-depleted Nb_4_N_3_ layer in the outer zone.

Analysis of Vickers microhardness measurements (Table 3) shows that the ceramic NbN coating with covalent bonds exhibits very high hardness, i.e., more than three times that of AISI 316L steel measured at 100 g and almost eight times that at a 50 g load. Oxidation of the niobium nitride coating resulted in a more than twofold reduction in hardness, while the microhardness values of such a coating are still higher compared to AISI 316L steel. The lower hardness of the oxidised coating is due to the high brittleness of the oxide layer formed on the surface. In the work of Fals et al. [31], the hardness of the Nb_2_O_5_ coating produced by thermal spraying was 633HV0.05. The result obtained is lower by almost 220HV0.05 compared to the oxide layer produced in the present work. The much thicker coatings (500 µm) produced by thermal spraying show porosity, which may contribute to a decrease in hardness.

**Figure 10:**
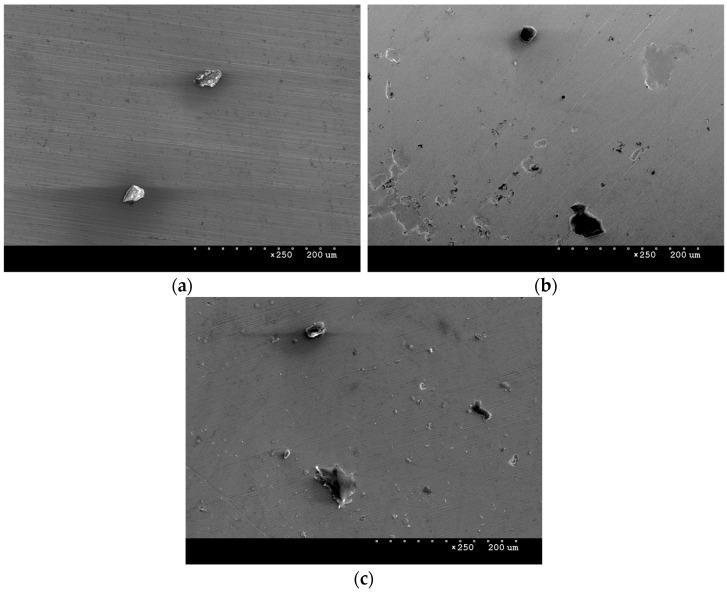
SEM images of the surface after bioactivity testing in SBF solution of samples: AISI 316L steel (**a**), NbN (**b**) and NbN + Nb_2_O_5_ (**c**) coatings.

The bioactivity of the produced NbN coating and Nb_2_O_5_ layers was investigated by microscopic observations on an SEM scanning electron microscope and by analysing the chemical composition of the samples exposed to an SBF solution. Figure 10 shows the surfaces of AISI 316L steel, NbN coating and oxidised NbN coating that were treated with SBF for 2 weeks. All surfaces showed the presence of phosphorus and calcium with numerous agglomerates, which was confirmed by chemical composition studies (Table 4). The elements were present on the entire surface of the samples, both in agglomerates and on surfaces where agglomerates were not observed. In addition to the percentages, the ratio of calcium to phosphorus was also determined, making it possible to identify whether calcium phosphates were formed on the surface. Hydroxyapatite may have been obtained on the NbN coating and oxidised NbN coating due to a similar Ca/P ratio (ratio for hydroxyapatite equal to 1.667) [32]. AISI 316L steel has the highest phosphorus content on the surface, which may indicate good bioactivity, but the Ca/P ratio was 1.2. In contrast, the closest ratio of Ca/P to hydroxyapatite was found for the NbN coating (1.73) but also for the NbN coating with produced niobium oxide Nb_2_O_5_, which showed a slightly higher value (1.77). The formation of calcium phosphates on the surfaces of both coatings with a Ca/P ratio close to 1.667 is, among other things, caused by the easier formation of phosphate layers on the ceramic surface (NbN, Nb_2_O_5_) than on the metallic surface (AISI 316L steel). Tests were carried out in several places on flat surfaces outside the visible agglomerates, which may indicate that thin layers of calcium phosphates were obtained across the surface of the samples. Further exposition of the tested materials in the SBF solution would probably lead to the formation of thicker, uniform layers of phosphates.

**Figure 11:**
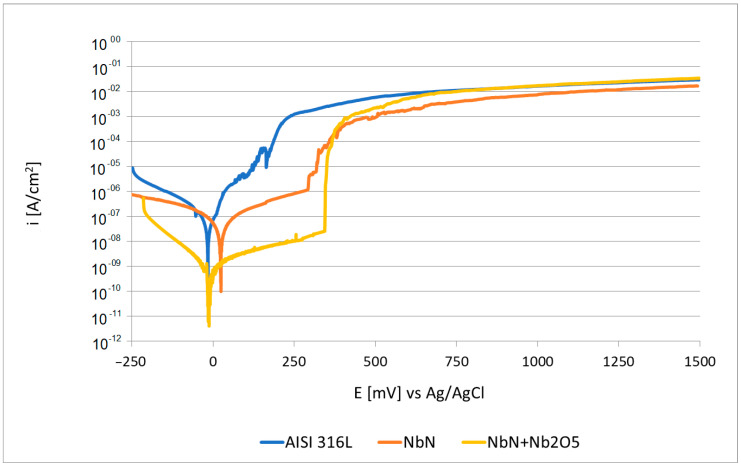
Anodic polarisation curves determined for AISI 316L initial state steel, and for NbN and NbN + Nb_2_O_5_ coatings in Ringer’s solution at 37 °C.

Potentiodynamic measurements of the tested materials (Figure 11) show that the NbN-coated steel had the highest corrosion potential, reaching a value of 10.8 mV (Table 5). Also, the oxidised NbN coating sample reached a higher corrosion potential (−20.8 mV) than the AISI 316L steel in its initial state (−34.6 mV). Interestingly, the lowest corrosion current density (6.16·10^−9^ A/cm^2^), the highest polarisation resistance (3.1·10^6^ Ω·cm^2^) and a high breakthrough potential of the passive layer (458 mV) were obtained for the ceramic niobium oxide Nb_2_O_5_ produced on the NbN coating, indicating its higher corrosion resistance compared to steel in its initial state. The NbN coating presented almost an equally high breakthrough potential (5 mV higher than the oxidised coating) but its polarisation resistance was an order of magnitude lower, with a corrosion current density two orders of magnitude higher than the oxidised coating. The better performance of the oxide coating may be mainly due to its higher dielectric properties compared to the nitride coating, which is associated with weaker electron exchange between the test material and the electrolyte during anodic polarisation. The reasons could also be attributed to the better quality of the NbN + Nb_2_O_5_ coating, which showed fewer discontinuities than the NbN coating, since the cavities present in the NbN coating were filled by the oxides during the oxidation process. It is also worth noting that, in the passive state, the NbN + Nb_2_O_5_ coating showed the lowest current densities (7.29·10^−9^ A/cm^2^ at a potential of 100 mV), while AISI 316L initial steel showed the highest (5.5·10^−6^ A/cm^2^). The unstable and narrow range of the passive state, as well as the high anodic current densities of the steel in the initial state, indicate the lowest durability of the passive layer formed on this material. The NbN coating had current densities in the passive state that were between those of the AISI 316L steel and the Nb_2_O_5_ layer (5.18·10^−7^ A/cm^2^) (Figure 11, Table 5).

The initial steel showed the lowest corrosion potential, the lowest polarisation resistance and the lowest breakthrough potential; therefore, it can be concluded that the NbN coating, as well as the oxidised NbN coating with an Nb_2_O_5_ top layer, improved the corrosion resistance of AISI 316L steel. Nb_2_O_5_ coatings with a nano-slice microstructure produced using a DC magnetron sputtering system in an argon–oxygen atmosphere on AISI 304 austenitic steel by Kumar et al. [33] exhibited significantly lower corrosion potentials and similar corrosion current densities and polarisation resistances determined in a 3.5% NaCl solution compared to the oxide layer produced in this work. On the other hand, Pillis et al. [34] produced an Nb_2_O_5_ coating by reactive DC magnetron sputtering from a niobium target in Ar + O_2_ atmosphere for 30 min, which was characterised by a corrosion current density of 36·10^−9^ A/cm^2^, a corrosion potential of −222 mV and a breakthrough potential of 210 mV determined in a 3.5% NaCl solution. The electrochemical parameters presented are inferior to those obtained in the present study; however, the authors also found a significant increase in the corrosion resistance of AISI 316 steel after the formation of an Nb_2_O_5_ coating.

**Figure 12:**
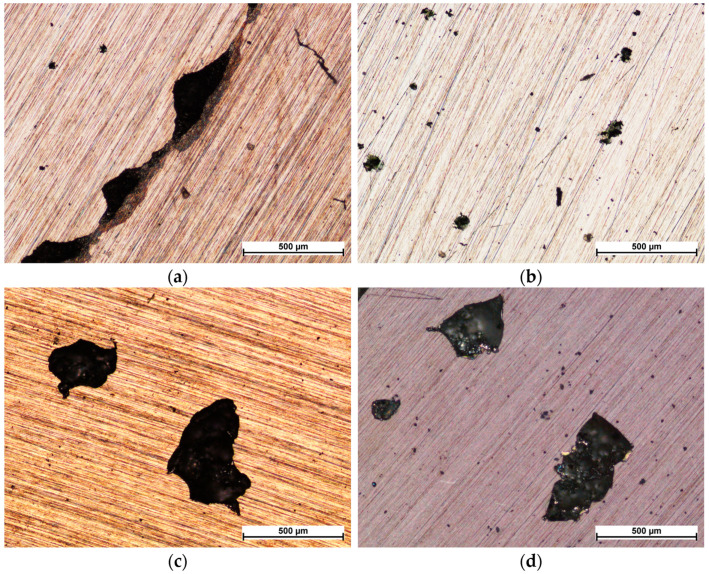
Surface after corrosion tests of AISI 316L steel in initial state (**a**,**b**), and with NbN (**c**) and NbN + Nb_2_O_5_ (**d**) coatings.

Based on microscopic observations of the tested materials after corrosion processes (Figure 12), it can be concluded that pitting corrosion appeared on the surface of each sample (Figure 12b–d), while crevice corrosion at the interface between the sample and the gasket occurred only on AISI 316L steel in its initial state (Figure 12a). The pitting on the surface of AISI 316L initial steel was smaller (Figure 12b) than on the produced coatings (Figure 12c,d); however, the amount of pitting was much higher compared to that which formed on the coatings. 

The larger pits in the NbN and NbN + Nb_2_O_5_ coatings may be the result of discontinuities visible under the SEM microscope (Figure 3 and Figure 4), where localised corrosion is initiated. On the other hand, pitting on initial steel forms in micro-areas, e.g., at inclusions, discontinuities and in mechanical flaws of the passive layer [19], resulted in the formation of a large number of smaller pits. Polarisation of the coated and uncoated samples at higher potentials (over 1500 mV) would lead to an increase in the size and number of pits.

**Figure 13:**
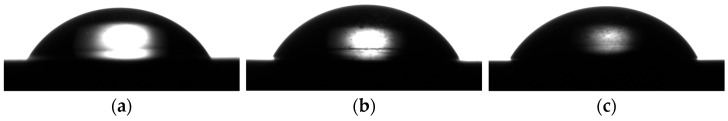
Shape of distilled water droplets on the surface of AISI 316L steel in initial state (**a**), and with NbN (**b**) and NbN + Nb_2_O_5_ (**c**) coatings.

Based on the droplet shapes shown in Figure 13 and the contact angle results shown in Table 6, it can be concluded that the best wettability is exhibited by the AISI 316L steel sample with an NbN coating and an Nb_2_O_5_ top layer, for which the contact angle reached the lowest value of 58.8° (Figure 13c). The tests showed a significantly higher contact angle of the NbN coating (Figure 13b), which was 70.5°, compared to initial steel (62.7°) (Figure 13a, Table 6). The surfaces of AISI 316L steel, NbN coating and NbN + Nb_2_O_5_ showed good wettability (θ < 90°), with a marked improvement in the hydrophilic properties of the oxidised NbN coating. The change in the wetting angle can be due to the coating’s surface roughness, as well as its chemical composition. Since high surface roughness (Table 2) usually increases hydrophobic properties [35], the hydrophilic properties of the coating with an Nb_2_O_5_ layer are mainly due to a change in the chemical composition of the tested surface and its high roughness plays a negligible role in this case.

Kumar et al. [33] obtained Nb_2_O_5_ coatings by using the sputtering technique in an Ar + O_2_ atmosphere. The coatings also showed hydrophilic properties; however, their contact angle was higher, ranging from 78.4 to 97.3°, depending on the deposition temperature, i.e., from room temperature to 200 °C. On the other hand, Pauline et al. [36], using the sol–gel method, produced Nb_2_O_5_ coatings on AISI 316L steel, which showed a contact angle of 75.8° measured in water, i.e., 17° more than the result obtained on the NbN coating with a Nb_2_O_5_ top layer. The results indicate that the oxidation process of the NbN coating makes it possible to obtain layers with very good hydrophilicity, which is important in terms of their application in biomaterials that come into contact with human tissue. Wettability significantly affects biocompatibility [37], including protein adsorption and thus cell adhesion to the material surface. Increased wettability contributes to increased cell adhesion to the biomaterial and thus may facilitate the process of permanent bonding of the implant to the patient’s bone [38].

## 4. Conclusions

- Calcium phosphates have formed on AISI 316L steel, as well as on NbN and NbN + Nb_2_O_5_ coatings after soaking in SBF solution, with the NbN coating and the oxidised NbN coating showing a Ca/P ratio closest to that of hydroxyapatite, which indicates good bioactivity of both the produced coatings.
- The sample with the oxidised NbN coating showed the best corrosion resistance in Ringer’s solution. Pitting corrosion was observed for each of the samples tested, while the AISI 316L sample without coating also showed crevice corrosion. For the NbN and the NbN + Nb_2_O_5_ coatings, a clear improvement in crevice corrosion resistance was observed.
- The formation of an Nb_2_O_5_ surface layer resulted in an increase in hydrophilic properties compared to the non-oxidised NbN coating. The contact angle of the oxidised coating was also lower compared to steel in its initial state, which can positively improve the osteointegration of bone tissue with the surface of the NbN + Nb_2_O_5_ coating.
- AISI 316L steel with an Nb_2_O_5_ top layer produced on NbN coating has the greatest potential for biomedical applications given its best combination of properties such as high corrosion resistance, bioactivity, surface hydrophilicity and increased hardness compared to AISI 316L steel.

## Figures and Tables

**Table 1:** Chemical composition of oxidised NbN coating measured in agglomerates and on flat surfaces of a layer.

Examined Area	N	O	Nb
	Weight %		
Agglomerates	2.1 ± 0.3	19.4 ± 2.1	78.5 ± 2.0
Flat surface	3.1 ± 0.1	15.5 ± 0.5	81.4 ± 0.5
	Atomic %		
Agglomerates	7.0 ± 1.1	54.3 ± 3.6	38.7 ± 2.9
Flat surface	10.7 ± 0.2	46.9 ± 0.8	42.4 ± 0.8

**Table 2:** Roughness measurements of samples: AISI 316L steel, NbN and oxidised NbN coatings.

Sample	Ra [nm]	Rt [nm]
AISI 316L	52 ± 1.3	1340 ± 49.9
NbN	108.6 ± 3.7	11,416.7 ± 775.4
NbN + oxidising	489.5 ± 12.3	17,146.7 ± 1507.8

**Table 3:** Vickers microhardness measurements of samples: AISI 316L steel, NbN and NbN + Nb_2_O_5_ coatings.

	AISI 316L	NbN	NbN + Nb_2_O_5_
HV0.1	290 ± 4	1046 ± 20	571 ± 10
HV0.05	296 ± 5	2256 ± 37	852 ± 10

**Table 4:** Percentage of elements on the surface of samples: AISI 316L steel, NbN and NbN + Nb_2_O_5_ coatings, subjected to bioactivity testing in SBF solution [% at.].

	Na [%]	Mg [%]	P [%]	Cl [%]	K [%]	Ca [%]	Ca/P
AISI 316L	0.11 ± 0.03	0.43 ± 0.12	1.80 ± 0.16	0.16 ± 0.05	0.06 ± 0.02	2.16 ± 0.19	1.20
NbN	0.06 ± 0.01	0.45 ± 0.13	1.30 ± 0.43	0.22 ± 0.07	0.03 ± 0.01	2.24 ± 0.25	1.73
NbN + Nb_2_O_5_	0.91 ± 0.21	0.45 ± 0.13	1.22 ± 0.36	0.61 ± 0.18	0.61 ± 0.15	2.17 ± 0.25	1.77

**Table 5:** Electrochemical parameters of AISI 316L steel, NbN and NbN + Nb_2_O_5_ coatings.

Sample	E_corr_ [mV]	i_corr_ [A·cm^2^]	R_pol_ [Ω·cm^2^]	I_pass_ * [A/cm^2^]	E_pit_ [mV]
AISI 316L	−34.6 ± 10.9	2.55·10^−7^ ± 9.7·10^−8^	1.97·10^5^ ± 4.56·10^4^	5.5·10^−6^ ± 1.68·10^−6^	243 ± 15
NbN	10.8 ± 5.8	2.48·10^−7^ ± 6.73·10^−8^	3.04·10^5^ ± 4.95·10^4^	5.18·10^−7^ ± 2.35·10^−7^	463 ± 38
NbN + Nb_2_O_5_	−20.8 ± 40.3	6.16·10^−9^ ± 1.93·10^−9^	3.10·10^6^ ± 5.90·10^5^	7.29·10^−9^ ± 2.56·10^−9^	458 ± 12

* Measured at 100 mV.

**Table 6:** Contact angle values of AISI 316L steel, NbN and NbN + Nb_2_O_5_ coatings.

Sample	AISI 316L	NbN	NbN + Nb_2_O_5_
Contact angle θ [°]	62.7 ± 1.9	70.5 ± 1.6	58.8 ± 1.3

## Data Availability

The data reported in this manuscript are available upon request from the corresponding author.

## References

[1] Wang W., Ji L., Li H., Zhou H., Chen J. (2017). Self-organized formation of nano-multilayer structure in the carbon-copper thin film during reactive magnetron sputtering deposition process. J. Alloys Compd..

[2] Zhou Z., Song Q., Xu Y., Liang H., Zhang M., Zhang B., Yan P. (2022). Magnetron sputtering deposited large-scale Weyl semimetal THz detector. Infrared Phys. Technol..

[3] Yang Q., Seo D.Y., Zhao L.R. (2004). Multilayered coatings with alternate pure Ti and TiN/CrN superlattice. Surf. Coat. Technol..

[4] Li Y., Xie M., Sun Q., Xu X., Fan X., Zhang G., Li H., Zhu M. (2019). The effect of atmosphere on the tribological behavior of magnetron sputtered MoS_2_ coatings. Surf. Coat. Technol..

[5] Pogrebnjak A., Smyrnova K., Bondar O. (2019). Nanocomposite Multilayer Binary Nitride Coatings Based on Transition and Refractory Metals: Structure and Properties. Coatings.

[6] Dawson-Elli D.F., Fung C.A., Nordman J.E. (1991). DC reactive magnetron sputtered NbN thin films prepared with and without hollow cathode enhancement. IEEE Trans. Magn..

[7] Ufuktepe Y., Farha A.H., Kimura S.I., Hajiri T., Imura K., Al Mamun M.A., Karadag F., Elmustafa A.A., Elsayed-Ali H.E. (2013). Superconducting niobium nitride thin films by reactive pulsed laser deposition. Thin Solid Film..

[8] Al Mamun M.A., Farha A.H., Ufuktepe Y., Elsayed-Ali H.E., Elmustafa A.A. (2015). Nanoindentation study of niobium nitride thin films on niobium fabricated by reactive pulsed laser deposition. Appl. Surf. Sci..

[9] Havey K.S., Zabinski J.S., Walck S.D. (1997). The chemistry, structure, and resulting wear properties of magnetron-sputtered NbN thin films. Thin Solid Film..

[10] Ezirmik K.V., Rouhi S. (2014). Influence of Cu additions on the mechanical and wear properties of NbN coatings. Surf. Coat. Technol..

[11] Ren Y., Jia J., Cao X., Zhang G., Ding Q. (2022). Effect of Ag contents on the microstructure and tribological behaviors of NbN–Ag coatings at elevated temperatures. Vacuum.

[12] Xu J., Peng S., Li Z., Jiang S., Xie Z.-H., Munroe P. (2021). The influence of semiconducting properties of passive films on the cavitation erosion resistance of a NbN nanoceramic coating. Ultrason. Sonochem..

[13] Stone D.S., Migas J., Martini A., Smith T., Muratore C., Voevodin A.A., Aouadi S.M. (2012). Adaptive NbN/Ag coatings for high temperature tribological applications. Surf. Coat. Technol..

[14] Hovsepian P.E., Ehiasarian A.P., Purandare Y.P., Biswas B., Pérez F.J., Lasanta M.I., de Miguel M.T., Illana A., Juez-Lorenzo M., Muelas R. (2016). Performance of HIPIMS deposited CrN/NbN nanostructured coatings exposed to 650 °C in pure steam environment. Mater. Chem. Phys..

[15] Purandare Y.P., Robinson G.L., Ehiasarian A.P., Hovsepian P.E. (2020). Investigation of High Power Impulse Magnetron Sputtering deposited nanoscale CrN/NbN multilayer coating for tribocorrosion resistance. Wear.

[16] Chen M., Ding J.C., Kwon S.-H., Wang Q., Zhang S. (2022). Corrosion resistance and conductivity of NbN-coated 316L stainless steel bipolar plates for proton exchange membrane fuel cells. Corros. Sci..

[17] Fonseca R.M., Soares R.B., Carvalho R.G., Tentardini E.K., Lins V.F.C., Castro M.M.R. (2019). Corrosion behavior of magnetron sputtered NbN and Nb1-xAlxN coatings on AISI 316L stainless steel. Surf. Coat. Technol..

[18] Huang W., Zalnezhad E., Musharavati F., Jahanshahi P. (2017). Investigation of the tribological and biomechanical properties of CrAlTiN and CrN/NbN coatings on SST 304. Ceram. Int..

[19] Borowski T. (2021). Enhancing the Corrosion Resistance of Austenitic Steel Using Active Screen Plasma Nitriding and Nitrocarburising. Materials.

[20] Wang L., Sun J., Sun J., Lv Y., Li S., Ji S., Wen Z. (2012). Niobium nitride modified AISI 304 stainless steel bipolar plate for proton exchange membrane fuel cell. J. Power Sources.

[21] Dinu M., Braic L., Padmanabhan S.C., Morris M.A., Titorencu I., Pruna V., Parau A., Romanchikova N., Petrik L.F., Vladescu A. (2020). Characterization of electron beam deposited Nb_2_O_5_ coatings for biomedical applications. J. Mech. Behav. Biomed. Mater..

[22] Moreto J.A., Gelamo R.V., Nascimento J.P.L., Taryba M., Fernandes J.C.S. (2021). Improving the corrosion protection of 2524-T3-Al alloy through reactive sputtering Nb_2_O_5_ coatings. Appl. Surf. Sci..

[23] Fals H.C., Belém M.J.X., Roca A.S., Fanton L., Lima C.R.C. (2019). Phase transformation of Nb2O5 during the formation of flame sprayed coatings and its influence on the adhesion strength, abrasive wear and slurry erosive wear. Wear.

[24] Nagarajan S., Raman V., Rajendran N. (2010). Synthesis and electrochemical characterization of porous niobium oxide coated 316L SS for orthopedic applications. Mater. Chem. Phys..

[25] Olivares-Navarrete R., Olaya J.J., Ramírez C., Rodil S.E. (2011). Biocompatibility of Niobium Coatings. Coatings.

[26] Eisenbarth E., Velten D., Breme J. (2007). Biomimetic implant coatings. Biomol. Eng..

[27] Johansson C.B., Albrektsson T. (1991). A removal torque and histomorphometric study of commercially pure niobium and titanium implants in rabbit bone. Clin. Oral Implant. Res..

[28] Hryniewicz T., Rokosz K., Sandim H.R.Z. (2012). SEM/EDX and XPS studies of niobium after electropolishing. Appl. Surf. Sci..

[29] Qi Z., Wu Z., Zhang D., Zuo J., Wang Z. (2016). Microstructure, mechanical properties and oxidation behaviors of magnetron sputtered NbNx coatings. J. Alloys Compd..

[30] Kokubo T., Takadama H. (2006). How useful is SBF in predicting in vivo bone bioactivity?. Biomaterials.

[31] Fals H.C., Lourençato L.A., Orozco M.S., Belém M.J.X., Lima C.R.C. (2020). Slurry erosion resistance of thermally sprayed Nb_2_O_5_ and Nb_2_O_5_ + WC12Co composite coatings deposited on AISI 1020 carbon steel. Ceram. Int..

[32] Ahmadi R., Izanloo S. (2022). Development of HAp/GO/Ag coating on 316 LVM implant for medical applications. J. Mech. Behav. Biomed. Mater..

[33] Kumar A., Malik G., Adalati R., Chawla V., Pandey M.K., Chandra R. (2021). Tuning the wettability of highly transparent Nb2O5 nano-sliced coatings to enhance anti-corrosion property. Mater. Sci. Semicond. Process..

[34] Pillis M.F., Geribola G.A., Scheidt G., de Araújo E.G., de Oliveira M.C.L., Antunes R.A. (2016). Corrosion of thin, magnetron sputtered Nb_2_O_5_ films. Corros. Sci..

[35] Yang S., Habazaki H., Fujii T., Aoki Y., Skeldon P., Thompson G.E. (2011). Control of morphology and surface wettability of anodic niobium oxide microcones formed in hot phosphate–glycerol electrolytes. Electrochim. Acta.

[36] Pauline S.A., Rajendran N. (2014). Biomimetic novel nanoporous niobium oxide coating for orthopaedic applications. Appl. Surf. Sci..

[37] van Kooten T.G., Schakenraad J.M., van der Mei H.C., Busscher H.J. (1992). Influence of substratum wettability on the strength of adhesion of human fibroblasts. Biomaterials.

[38] Jung U.-W., Hwang J.-W., Choi D.-Y., Hu K.-S., Kwon M.-K., Choi S.-H., Kim H.-J. (2012). Surface characteristics of a novel hydroxyapatite-coated dental implant. J. Periodontal Implant. Sci..

